# Avoiding Pain to Others Motivates Effortful Prosocial Behavior

**DOI:** 10.1111/nyas.70075

**Published:** 2025-10-01

**Authors:** Claudia Massaccesi, Lei Zhang, Giorgia Silani, Claus Lamm

**Affiliations:** ^1^ Department of Cognition, Emotion, and Methods in Psychology, Faculty of Psychology University of Vienna Vienna Austria; ^2^ Department of Psychology, Section for Biological Psychology and Cognitive Neuroscience University of Bielefeld Bielefeld Germany; ^3^ Centre for Human Brain Health, School of Psychology University of Birmingham Birmingham UK; ^4^ Institute for Mental Health, School of Psychology University of Birmingham Birmingham UK; ^5^ Centre for Developmental Science, School of Psychology University of Birmingham Birmingham UK; ^6^ Department of Clinical and Health Psychology, Faculty of Psychology University of Vienna Vienna Austria

**Keywords:** effort‐based decision‐making, effort discounting, harm avoidance, pain, prosocial behavior, social decision‐making

## Abstract

Protecting others from harm is critical for societal well‐being, but is often effortful. How individuals weigh the costs of exerting effort against the benefits of avoiding harm to others is currently unknown. To fill this gap, we investigated how individuals decide to exert physical effort to reduce painful shocks delivered to themselves and another person. Results showed that individuals are similarly motivated to incur effort costs to reduce their pain and the pain of another person. Specifically, we found no credible evidence that participants’ willingness to put in effort and the force they exerted to reduce pain differed when helping the other person versus themselves. Further, we showed little credible evidence of a difference in discounting of pain reduction by effort between self‐ and other‐related choices. These results contrast with prior research indicating that individuals are less motivated to exert effort to gain (or avoid losing) monetary rewards for others than themselves and demonstrate that protecting others from harm shifts individuals’ effortful behavior from prosocially apathetic to prosocially motivated. Our findings shed light on the motivational processes underlying interpersonal harm avoidance and effortful prosocial behavior and highlight the importance of the type of benefit at stake for motivating prosociality.

## Graphical abstract

Protecting others from harm is critical for societal well‐being but is often effortful. We examined how individuals choose to exert physical effort to reduce their and another person's pain. Results showed that individuals are similarly motivated to incur effort costs to help themselves and another person. We demonstrated that humans are not inherently selfish when effort is the cost of acting prosocially, as suggested by prior research, and that protecting others from physical harm promotes prosociality.

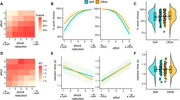

## 1 Introduction

Prosocial acts, like caring or helping, aiming at reducing or preventing another person's suffering, are often effortful. In recent years, research on prosocial behavior has begun to unravel the cognitive and neural mechanisms underlying how and why we decide to exert (physical and cognitive) effort to benefit another person [1]. Our current understanding of how effort shapes prosociality is based predominantly on studies using monetary incentives, where the prosocial act is directed toward benefiting others financially. Much less is known about the relationship between effort costs and prosociality in the context of non‐monetary incentives, such as harm avoidance. The present study aimed to fill this knowledge gap and examined how individuals decide to exert physical effort to protect others from pain.

Over the past four decades, research on prosociality has consistently shown that, contrary to the neoclassical view of the Homo Economicus, a rational and self‐interested agent, individuals are willing to sacrifice personal interests to benefit others, even strangers [2, 3]. However, research has also revealed that, despite this willingness to share and help, individuals still value their own wealth more than that of others [2, 4]. For example, in a classic dictator game, most individuals endowed with money share a portion of their endowment with another individual. Yet, people usually do not split their monetary resources equally but keep a larger part of the endowment to themselves [2]. Consistently, individuals can learn to gain monetary rewards (or avoid monetary losses) for others, but their learning rate is lower when benefitting others compared to themselves [5−8], but see Ref. [9]. In this kind of learning tasks, lower learning rates are commonly less optimal [10], further demonstrating a self‐benefitting or “egocentric” asymmetry in prosocial decision‐making. Moreover, when benefitting someone financially requires exerting physical effort, individuals are prosocially apathetic, discounting monetary rewards by effort more strongly for others than themselves, and prosocially superficial, exerting less force to benefit others than themselves [11−15]. Similar findings have been reported for tasks requiring cognitive effort and in the case of avoiding others’ monetary loss [13, 16]. This egocentric bias is amplified by stress [17]. Given that stress promotes automatic, intuitive responses [18, 19], this suggests that humans are inherently selfish when the benefit at stake is of a financial nature.

While investigating prosociality using monetary incentives has been a useful standard in experimental psychology and neuroeconomic research, many everyday prosocial acts happen in non‐financial contexts, like those aimed at preventing others’ harm. Interestingly, while studies using monetary incentives indicate a bias toward selfish behavior, those involving reducing others’ harm (e.g., pain) revealed exceptional altruistic tendencies. For example, research showed that individuals greatly value protecting others’ physical well‐being, as seen in tasks where participants risk their (virtual) lives, endure pain, or sacrifice money to prevent harm to another person [20−22]. Notably, individuals prioritize reducing others’ pain over their own as they are willing to forego more money to protect others from pain than to protect themselves [12, 23−26]. However, they are egoistic when they need to harm themselves to gain monetary rewards for others [27]. Further, using a modified dictator game involving the allocation of money (gains and losses) and pain, two studies showed that individuals are significantly more prosocial in the pain versus the monetary context [27, 28]. Last, contrary to what was observed in financial contexts, we recently showed that individuals learn more optimally to avoid painful shocks directed at a stranger than shocks directed at themselves [29]. Taken together, these findings indicate that others’ physical well‐being strongly influences human prosocial behavior and suggest that protecting others from harm holds greater motivational value than increasing others’ wealth. However, prior studies examining prosociality in the context of harm avoidance exclusively assessed choice behavior, neglecting effortful action invigoration, and involved mainly monetary costs. We recently showed that individuals are willing to incur effort costs to avoid pain to others and that this motivation to help is reduced by placebo analgesia [30]. This study, however, did not include a condition of self‐beneficial effort. Thus, whether individuals show a selfish or a prosocial bias when avoiding harm requires initiation of effortful acts and the actual exertion of physical effort is currently unknown.

Here, we address this knowledge gap and aim to demonstrate that avoiding others’ physical harm is a strong driver of effortful prosociality and that effort costs do not necessarily lead to prosocial apathy or superficiality. To this end, we used a novel adaptation of a previously established prosocial effort task [13, 30]. Participants (*N* = 50) were asked to decide whether they would exert physical effort to reduce the number of painful shocks delivered to their own hand or to that of another participant. If they chose to exert effort, they needed to apply actual physical force by squeezing a hand‐dynamometer. We assessed prosocial motivation using both mixed‐effect statistical models and cognitive computational modeling to compare subjective value computations underlying the willingness to exert effort and the amount of force exerted to reduce one's pain or another person's pain. We aimed to clarify whether humans are inherently selfish when effort is the cost of acting prosocially, as suggested by prior research, or if protecting others from physical harm promotes prosociality, shifting effortful behavior from self‐centered to other‐centered.

## 2 Materials and Methods

### 2.1 Participants

A total of 53 participants took part in the study. Eligibility criteria included being between 18 and 40 years old, no history of current or former neurological conditions or psychiatric disorders, no regular intake of psychotropic medications, no history of current or former substance abuse, and fluency in German or English. The sample size was determined based on an *a priori* power analysis for repeated‐measure *F*‐tests performed with G*Power [31], showing that a sample size of 46 would provide 90% power to detect an effect size of Cohen's *f* = 0.2 at alpha level 0.05 (effect size estimate was based on previous studies on prosocial behavior in harm avoidance and studies using a similar prosocial effort task [13, 15, 23]). Psychology students were excluded due to possible familiarity with the task and the deceptions involved. Three participants were excluded as they did not comply with the eligibility criteria: one participant was found to have insufficient English and German comprehension during the testing phase, and two participants revealed to be under treatment with psychotropic medications at the post‐test questionnaire. The final sample thus included 50 participants (28 females; age: M = 25.4, SD = 4.3, range = 18–36). Participants were recruited using the Vienna CogSciHub: Study Participant Platform, which uses the hroot software [32]. The study was conducted in line with the Declaration of Helsinki and approved by the Ethics Committee of the University of Vienna. Participants signed a consent form before participation and were debriefed regarding the deceptions at the end of the study.

### 2.2 Prosocial Effort Task

**FIGURE 1:**
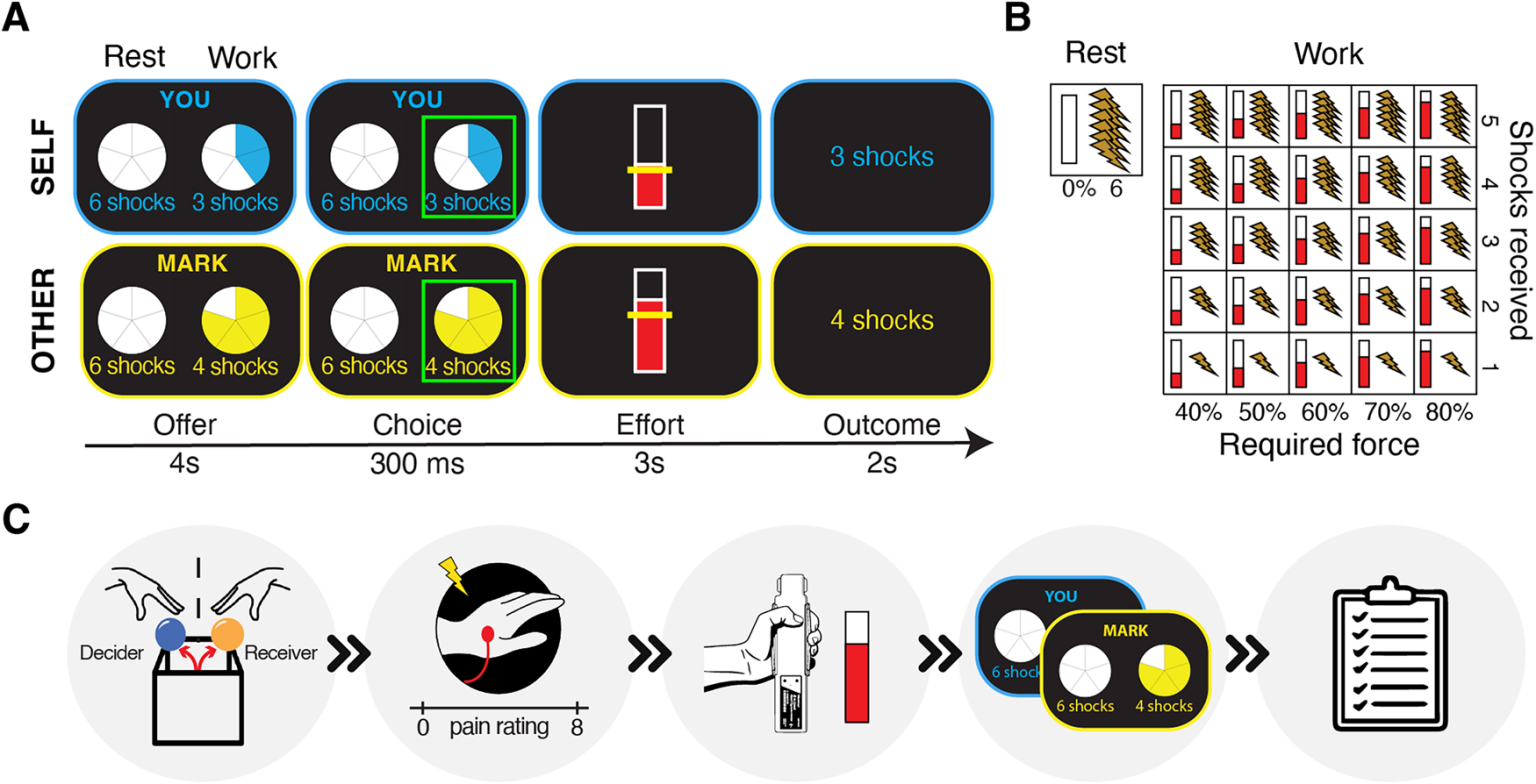
Trial structure of the prosocial effort task and overview of the study procedure. (A) Each trial of the prosocial effort task presents a choice between a rest offer (no effort, six shocks) and a work offer (variable effort, variable shock reduction). If the work offer is chosen, participants have to exert the required amount of effort (by squeezing a hand‐dynamometer) to achieve the shock reduction. In half of the trials, participants choose for themselves (self), and in the other half, they choose for the other (other). (B) The rest option is associated with no effort (0%) and six shocks. The work option entails combinations of effort (40%, 50%, 60%, 70%, or 80% of the participants’ maximum voluntary contraction) and shocks (1–5). (C) Overview of the study procedure: (1) participants and confederates were assigned the role of decider and receiver, respectively, using a putatively random role assignment procedure, (2) participants’ individual pain threshold and maximum voluntary contraction were estimated, (3) participants performed the prosocial effort‐task, and (4) completed post‐test questionnaires.

We developed a novel version of the prosocial effort task adapted from Hartmann et al. [30] and Lockwood et al. [13], in which participants are required to exert effort to avoid physical pain to themselves and another person. In each trial of the task (Figure 1A), participants could choose between a “rest offer” of receiving six electric shocks for no effort and a “work offer” of receiving a variable lower amount of shocks (1, 2, 3, 4, or 5) for exerting a variable amount of effort (40%, 50%, 60%, 70%, or 80% of the participant's maximum voluntary contraction [MVC]) (Figure 1B). When choosing the “work offer,” participants were required to exert the indicated amount of effort by squeezing a hand‐dynamometer with their dominant hand. Participants had to reach the required level of effort for at least 1 s out of a 3‐s window to attain the shock reduction. If participants did not make a choice within 4 s or did not reach the required level of effort, 10 shocks were assigned. Importantly, in half of the trials, participants made choices to avoid shocks directed at their hand (“self”), while in the other half, to avoid shocks directed at another participant (“other”; the confederate, see *Procedure* for details). Trial order was pseudo‐randomized (mini blocks of five randomized effort levels, so that the average effort level over the preceding five trials was always 2–3, and the same recipient was not presented more than four times consecutively). All trials had the same duration regardless of the choice made.

To avoid high‐effort offers being perceived as risky, before starting the task, participants experienced all effort levels to ensure they could attain the benefits at each level [1]. For each of the five effort levels, they were asked to complete two items from the NASA Task Load Index [33] (Physical Demand: “How physically demanding was the task?”, Effort: “How hard did you have to work to accomplish your level of performance?”) and one additional question (Unpleasantness: “How unpleasant was it for you?”), using a visual analogue scale from 0 (not at all) to 100 (very much). Participants performed 150 trials, 75 with outcomes for themselves (“self” trials) and 75 with outcomes for the other (“other” trials), with self‐paced breaks every 50 trials to avoid fatigue. Participants were informed that no shocks would be delivered during the task and that three trials from the “self” and three from the “other” condition would be randomly selected and executed at the end of the task.

### 2.3 Procedure

At the beginning of the study, a role assignment procedure was conducted to (putatively) randomly assign the role of “decider” (who would make decisions during the prosocial task) and “receiver” (who would receive the outcome of the decisions) among the two participants [13, 23, 30]. Participants were informed they would participate in the study together with another person (in reality, a gender‐matched confederate), who, however, they would not meet in person to limit the effects of reputation and reciprocity and to ensure anonymity (except for knowing each other's name). The participant and the confederate were positioned on either side of a door, wearing gloves to avoid revealing their identity, and they drew a ball from a box that revealed their role (Figure 1C). The participant was always assigned the role of “decider” and the confederate of “receiver”.

Then, participants’ pain threshold and MVC were estimated (Figure 1C). For the pain calibration, electrical stimulations were administered to the back of the non‐dominant hand using a Digitimer DS5 Isolated Bipolar Constant Current Stimulator (Digitimer Ltd, Clinical & Biomedical Research Instruments) to gain average stimulation intensities between 1 (perceivable, but not painful) and 8 (extremely painful) and to identify the stimulus intensity to be used in the prosocial effort task, corresponding to a level of 7. Participants’ MVC was determined by asking them to squeeze the hand‐dynamometer with their dominant hand with as much force as possible three consecutive times. The greatest force exerted was used to individually calibrate the effort levels in the prosocial effort task.

Following these procedures, participants performed the prosocial effort task. After the task, three trials were (allegedly) randomly selected and paid out (i.e., the shocks were delivered) to each recipient (Supplementary Materials). In reality, trials with the same three outputs were always selected for both recipients (1, 2, and 5 shocks, presented in a randomized fashion). In reality, the confederate never received any shock. After each outcome was paid out, the participant was asked to rate the painfulness and unpleasantness of the shocks received by themselves (“How painful were these shocks for you?”; “How unpleasant was it for you to receive these shocks?”) and by the confederate (“How painful were these shocks for the other participant?”; “How unpleasant was it for you when the other participant received these shocks?”), on a 7‐point Likert scale (1 = not at all, 7 = extremely). Last, participants filled out a series of questionnaires regarding sociodemographic information, traits of empathy via the Questionnaire of Cognitive and Affective Empathy (QCEA) [34] and helping attitudes via the Helping Attitudes Scale (HAS) [35], and quality control questions (assessing whether participants believed their anonymity was kept and their decisions were kept secret from the other participant, and whether they felt their decisions were monitored by the experimenters, plus an open comment on the other person).

### 2.4 Statistical Analysis

Analyses were performed in R (version 4.3.0) [36]. We assessed differences between “self” and “other” in choice behavior, reaction time (RT), force exerted, and success rate using generalized linear mixed‐effects models (gLMM; binomial model in the case of choice behavior). Models were fitted using the glmmTMB() function of the glmmTMB R package [37]. Choice behavior was coded as a binomial variable (0 = rest offer; 1 = work offer). Force exerted was computed as the area under the curve (AUC) for the 3‐s window in which participants exerted force, normalized as a proportion of their MVC. No data were discarded due to exceptionally fast RTs (only two trials in the entire dataset had RTs below 300 ms). We initially fitted maximal models including fixed and random main and interaction effects of recipient, z‐scored effort, z‐scored shock reduction, and by‐subject random intercept. In case of model non‐convergence or singularity, random slopes with the lowest cumulative variance were removed (see Tables S1−S4 for model specification). We analyzed ratings of shocks’ painfulness and unpleasantness using two gLMMs, including fixed and random main and interaction effects of recipient and number of shocks (1, 2, and 5), and by‐subject random intercept.

Null findings were followed up with Bayesian paired‐sample *t*‐tests (self vs. other) in JASP 0.16.4 [38] to compute Bayes factors (BF01, contrasting the null against the alternative hypothesis). We used the default Cauchy prior (*r* scale prior width = 0.707). A BF01 between 3 and 10 is considered moderate, and a BF01 larger than 10 is considered strong evidence for the null hypothesis. In contrast, a BF01 between 0.3 and 0.1 is considered moderate, and a BF01 smaller than 0.1 is considered strong evidence for the alternative hypothesis [39, 40].

We used Pearson correlations to test for associations between the task variables (choice, force, discounting parameter), the ratings of shocks’ painfulness and unpleasantness, and the trait scores of empathy (QCEA), and attitudes to help (HAS). We conducted control analyses on participants’ beliefs about anonymity and feelings of being monitored and on the effects of fatigue (results in Supplementary Materials). Six participants expressed doubts regarding the existence of the other person. Removing them from the gLMMs did not change the results’ pattern, unless differently stated in the Results section.

### 2.5 Hierarchical Bayesian Modeling

To formally quantify latent trial‐by‐trial cognitive mechanisms underlying choice behavior in the prosocial effort task, we tested established computational models of effort discounting [41−43], including single or separate components for self and other [13, 17]. All models assumed that participants would compute a subjective value (SV) for work offers, with levels of shock reduction being discounted by effort. These models differed in their way of specifying the shape of the discount function (κ) of SV at the trial (t) level: linear [SV = Shock_(t)_ + κ*Effort_(t)_], parabolic [SV = Shock_(t)_ + κ*Effort_(t)_^2^], or hyperbolic [SV = Shock_(t)_ * (1 / (1 + κ*Effort_(t)_))]. Shock was coded as −5 to −1, Effort as 1 to 5, and the SV of the “rest offer” was always −6. We coded “Shock” with negative values, rather than coding it as “shock reduction” with positive values, to best reflect the nature of aversive harm in our experimental design. Accordingly, all κ parameters were also negative. To facilitate interpretation, however, the extracted κ values were transformed to their absolute value. Higher κ values reflect greater effort discounting, whereas lower κ values indicate less effort discounting. All models also included the softmax parameter β, which defines the stochasticity or “noise” of each participant's choices. Lower β values reflect more stochastic behavior, while higher β values indicate more deterministic choices. We compared models with single and separate noise (β) and discount (κ) parameters for self and other to test the computational hypotheses: 1κ1β, 2κ1β, 1κ2β, 2κ2β.

#### 2.5.1 Model Estimation and Selection

Model estimation was performed with the hierarchical Bayesian approach [44] using Stan [45] implemented in R [36], following best practices from the hBayesDM R package [46]. We fitted each candidate model with four independent Markov Chain Monte Carlo chains using 1000 iterations after initial 1000 iterations for the algorithm fine‐tuning per chain, which resulted in 4000 valid posterior iterations (i.e., samples). Data from the self and other conditions were fitted concurrently with effect coding (e.g., κ_OTHER_ = κ_SELF_ + κ_EFFECT_) to account for within‐subject dependencies. The effect coding approach has been successfully applied in the past to accurately capture statistical variances of within‐subject designs [47, 48]. All group‐level parameters had a prior normal distribution, Normal (0, 1). Non‐response trials (self = 0.50% and other = 0.75% on average per subject) were excluded. Three participants were excluded from the modeling analyses as they displayed extreme choice behavior (almost exclusively choosing the work offer only for other and rarely for self, or the reverse), leading to *^R* values greater than 1.05 (Figure S4). The computational modeling analyses were thus conducted on 47 participants. Following the removal of these participants, all fitted models converged with *^R* < 1.01 [49].

Models were compared using the Leave‐One‐Out information criterion (LOOIC) computed with the *loo* R package [50]. A lower LOOIC score indicates better out‐of‐sample prediction accuracy of the candidate model after controlling for potential overfitting. Model‐predicted discounting (κ) of self and other from the winning model were extracted and compared using a paired *t*‐test.

#### 2.5.2 Model Validation and Parameter Recovery

We conducted a model validation (i.e., posterior predictive check) and parameter recovery analysis on data simulated by the winning model from synthetic participants. This synthetic dataset was generated by freely sampling individual parameters from the posterior group‐level ground truth values extracted from the winning model. First, we simulated choice rates, to examine whether the simulated data accurately reflected the main features of participants’ behavior, that is, whether choices vary as a function of effort and shock reduction (Figure S1). We then evaluated group‐level parameter recovery by verifying if the 95% highest density interval (HDI) of the true group‐level parameter distribution included mean estimates of the simulated group‐level parameter values (Figure S2A). Last, we evaluated subject‐level parameter recovery by calculating the correlation between the simulated and true estimated subject‐level parameters (Figures 3B and S2B).

**FIGURE 2:**
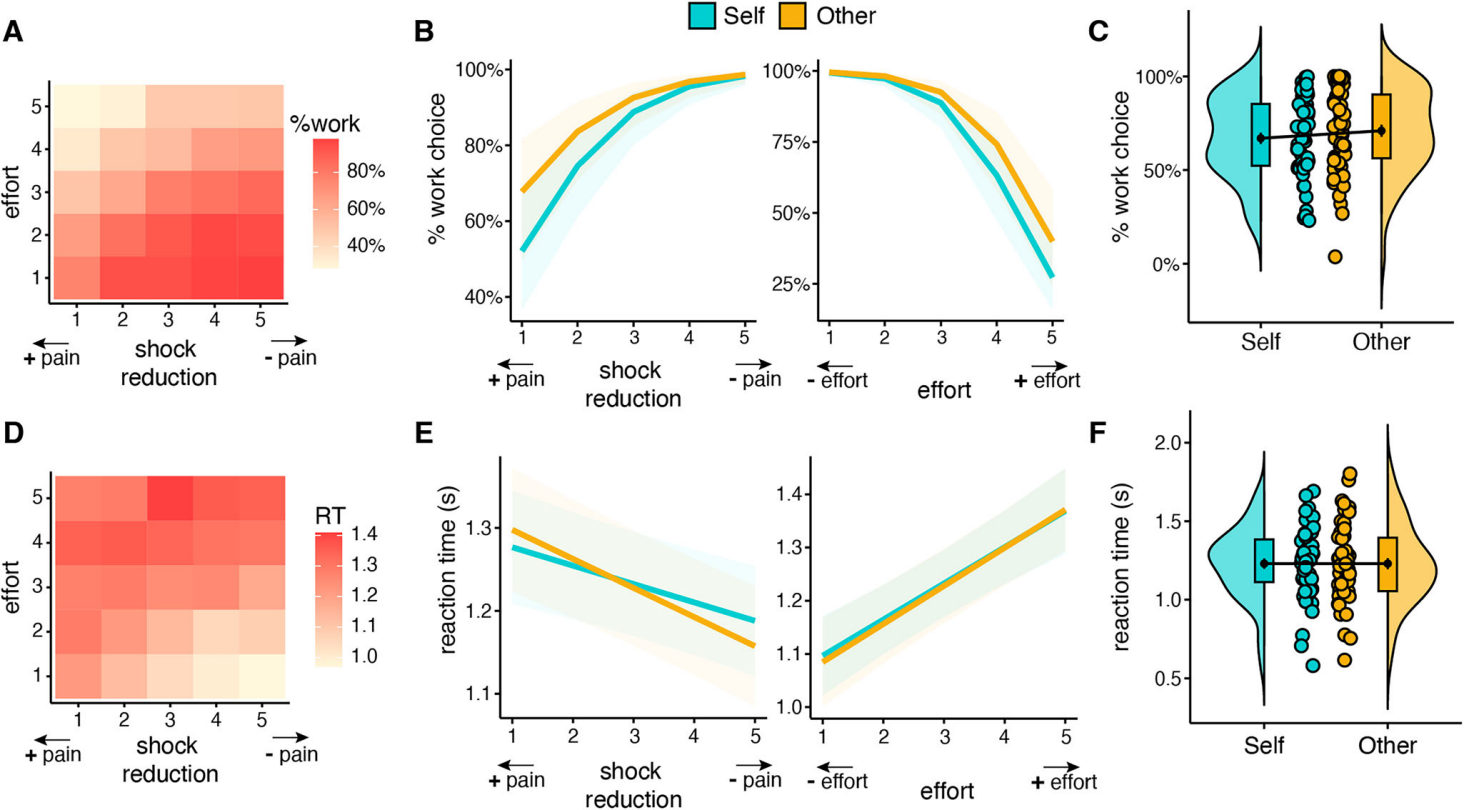
Choice behavior and reaction times in the prosocial effort task. Heatmap showing the percentage of chosen work offers (A) and reaction times (D) as a function of effort and shock reduction. (B) Percentage of chosen work offers and (E) reaction times as a function of recipient, shock reduction (left panel), and effort (right panel). The percentage of work choices decreased as the effort required increased, and it increased with greater shock reduction, similarly for self and other. Reaction times were longer for decisions involving greater effort and lower shock reduction, similarly for self and other. Participants did not significantly differ in their average (C) percentage of chosen work offers and (F) reaction times for self and other. The plots C and F depict the data distributions (half violins), boxplots, group means including the standard error of the mean (black points), and individual means (colored points).

### 2.6 Data and Code Availability

Data and code to support and reproduce the main findings of the study are openly available in OSF at https://osf.io/dxawv/.

## 3 Results

### 3.1 Individuals Are Not Prosocially Apathetic When Choosing to Exert Effort to Reduce Others’ Pain

We first assessed whether participants were more willing to exert effort to reduce pain to themselves or to the other participant. We fit a gLMM on participant's choices to rest or work, which revealed a significant Effort × Shock reduction interaction (OR = 0.67, *Cohen's d* = −0.22, CI [0.58, 0.78], *p* < 0.001) (Table S1). Higher shock reduction and lower effort were associated with increased work choices (Figure 2A). The fact that effort costs and the outcome (i.e., reduction of shocks) influenced participant choices indicates that the task worked as expected. Importantly, we observed no significant effects of recipient (self, other), indicating that participants were equally willing to exert effort to reduce their own pain (67% work choices) and the other's pain (71% work choices) (all *p* > 0.15, Table S1 and Figure 2B,C). Results were further confirmed by Bayesian statistics, showing substantial evidence in support of the null hypothesis (BF01 = 4.40). Moreover, participants’ willingness to exert effort to reduce the other participant's pain was not significantly correlated with trait cognitive (*r* = 0.01, *t*(48) = 009, *p* = 0.93, BF01 = 5.65) and affective (*r* = −0.07, *t*(48) = −0.51, *p* = 0.61, BF01 = 5.36) empathy, nor with their attitudes to helping (*r* = 0.25, *t*(48) = 1.80, *p* = 0.08, BF01 = 1.25).

As previous research suggested that individuals are slower when deciding to incur costs to benefit another person compared to themselves [15, 23, 24], we examined whether RTs differed in self and other trials (Table S2). We found that participants decided faster when the work offer involved high reward (i.e., high reduction of shocks) and low effort cost, and slower when the work offer involved high/medium reward and high effort cost (Effort × Shock reduction: b = 0.09, CI [0.06, 0.13], *p* < 0.001; Figure 2D,E). The gLMM also showed a significant Recipient × Shock reduction interaction (b = −0.04, CI [−0.07, 0.00], *p* = 0.046; Figure 2E), which became not significant following the exclusion of the six participants who expressed doubts regarding the cover story (*p* = 0.061). We found no other significant main or interaction effect of Recipient, indicating that participants were not slower when deciding whether to exert effort to help the other person (all *p* > 0.51; RT self: M = 1.23, SD = 0.23; RT other: M = 1.23, SD = 0.25, Table S2 and Figure 2F). The absence of a significant difference in RTs for self and other was confirmed by Bayesian statistics, indicating substantial evidence in support of the null hypothesis (BF01 = 6.22).

Overall, these results indicate that participants are not prosocially apathetic, both in terms of choice behavior and speed of choice, when effort is required to help someone in pain.

### 3.2 Individuals Are Not Prosocially Superficial When Exerting Force to Reduce Others’ Pain

**FIGURE 3:**
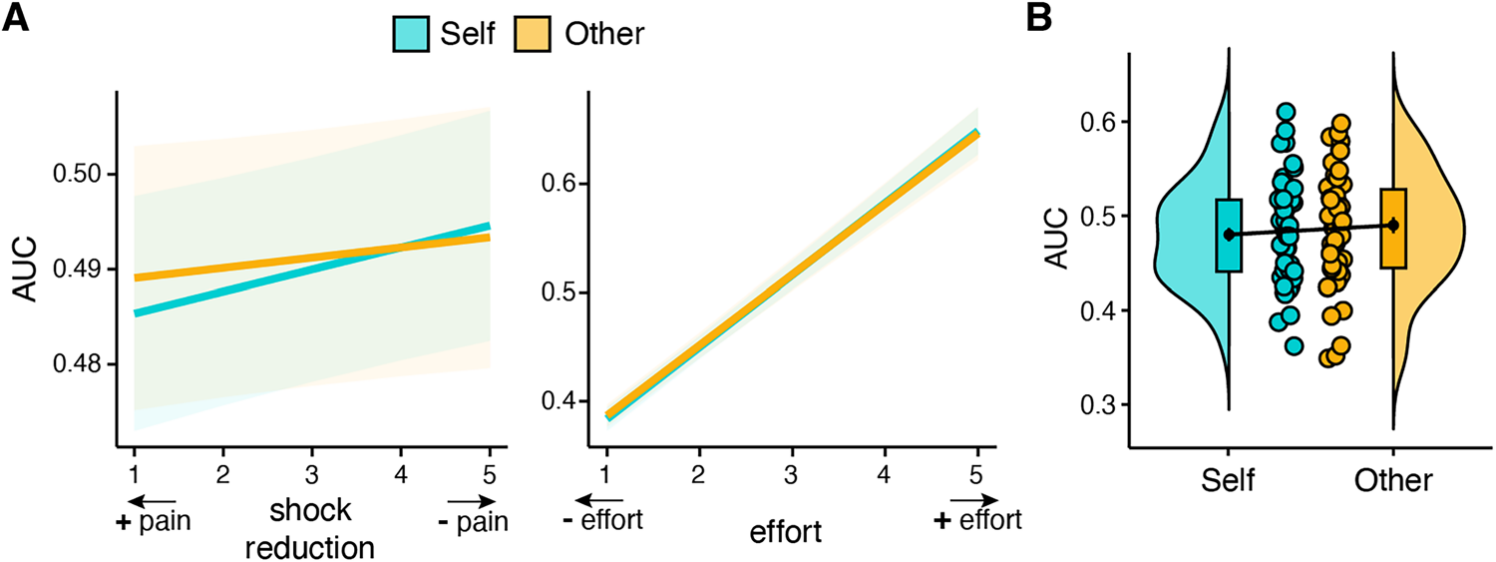
Force exerted in the prosocial effort task. (A) Force exerted (area under the curve, AUC) as a function of recipient, shock reduction (left panel), and required effort (right panel). Force exerted increased as the effort required and the amount of shock reduction increased, similarly for self and other. (B) Participants did not significantly differ in the average force they exerted for themselves and for the other. The plot depicts the data distributions (half violins), boxplots, group means including the standard error of the mean (black points), and individual means (colored points).

Previous research showed that even when individuals choose to mobilize effort to benefit another person, they are prosocially superficial, that is, they exert less force for others, particularly when effort costs are high [13, 15]. We thus examined whether participants exerted less force (expressed as area under the curve) to reduce the other participant's pain compared to their own (Table S3). As expected, we found that participants exerted more force when the effort required was higher (b = 0.74, CI [0.68, 0.81], *p* < 0.001) and when the shock reduction was higher (b = 0.03, CI [0.01, 0.05], *p* = 0.015) (Figure 3A). Importantly, no significant main or interaction effects of recipient were observed (all *p* > 0.27), indicating that participants’ force exerted did not differ in self and other trials, as they exerted a similar amount of force to benefit themselves and the other participant (self: M = 0.48, SD = 0.05; other: M = 0.49; SD = 0.06; Figure 3A,B). The absence of a significant difference in the force exerted for self and other was confirmed by Bayesian statistics, showing substantial evidence in support of the null hypothesis (BF01 = 4.24). Moreover, participants’ force exerted to reduce the other's pain was not significantly correlated with trait cognitive (*r* = −0.04, *t*(48) = −0.27, *p* = 0.79, BF01 = 5.47) and affective (*r* = −0.07, *t*(48) = −0.51, *p* = 0.61, BF01 = 5.00) empathy, nor with their attitudes to helping (*r* = 0.24, *t*(48) = 1.69, *p* = 0.10, BF01 = 1.50).

We also examined participants’ success rates in the trials in which they decided to exert effort to benefit themselves and the other participants. In line with what was reported for the force exerted, the success rate did not significantly differ in self (93%) and other (92%) trials (BF01 = 4.56; Table S4).

Overall, these results indicate that participants are not prosocially superficial when exerting effort to reduce another person's pain.

### 3.3 Pain Reduction for Self and Other is Discounted by Effort at a Similar Rate

**FIGURE 4:**
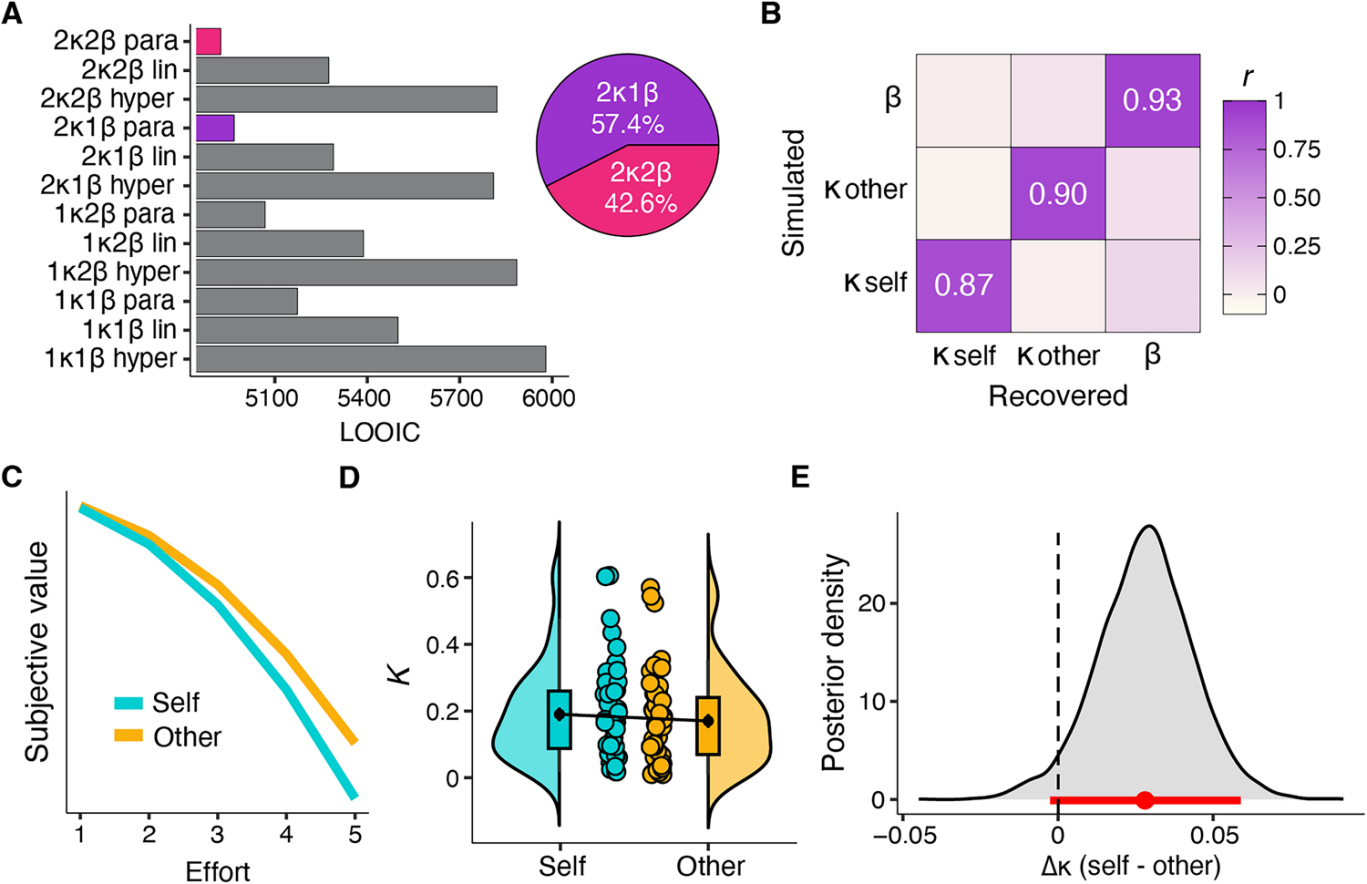
Hierarchical Bayesian modeling. (A) Leave‐One‐Out information criterion (LOOIC) values (bars; lower values indicate better fit) from the comparison of the computational models of effort discounting. The pie chart shows the number of participants with the lowest LOOIC for model 2κ1β and model 2κ2β. Model 2κ1β was selected as the winning model. (B) Parameter recovery of subject‐level discounting (κ self and κ other) and noise (β) parameters. The correlation matrix shows the Pearson's correlation between the actual recovered and simulated data from the winning model. The high correlations (all *r* > 0.80) indicate excellent parameter recovery. (C) Subjective value as a function of effort and recipient. (D) Discounting parameter (κ) as a function of recipient (self and other). The plot depicts the data distributions (half violins), boxplots, group means including the standard error of the mean (black points), and individual means (colored points). (E) Posterior distribution of the difference in the discounting parameter (Δκ) between self and other. The red point and line represent the mean and 95% highest density interval, respectively.

At the group level, the model 2κ2β parabolic had the lowest LOOIC (4967.529), followed closely by the model 2κ1β parabolic (4924.337), indicating similar predictive performance overall (Figure 4A). We thus examined the individual‐level LOOIC values for each participant to determine the proportion of participants for whom each model provided the best fit. Model 2κ1β was the best‐fitting model for 57% of participants, whereas model 2κ2β was the best‐fitting model for 42% (Figure 4A). Moreover, the posterior distributions of the parameters in 2κ2β revealed significant overlap between the two β parameters for self and other, suggesting that these parameters do not differ statistically (Figure S3C). Given the minimal improvement in group‐level LOOIC from 2κ2β, the lack of statistical differentiation between the β parameters for self and other, the broader applicability of the model 2κ1β across participants, and the more parsimonious explanation of the data with only one β parameter, we selected the 2κ1β as the winning model on which we based our further analyses on. Model validation and parameter recovery confirmed that the model accurately reproduced core aspects of the actual choice data (Figure S1) and that individual‐ and group‐level parameters can be successfully recovered (Figures 4B and S2). For completeness, we conducted model validation and parameter recovery also for the model 2κ2β (results reported in Supplementary Materials; Figures S1 and S3).

Overall, participants’ choice behavior was captured best by the model with a parabolic discounting function including separate κ for self and other and a single noise parameter (β), consistent with previous findings in the monetary context [13, 15, 43]. We compared the κ for self and other estimated from the winning model to examine differences in devaluation of shock reduction by effort in the two conditions. Effort discounting did not significantly differ between self and other trials (*t*(46) = −1.47, *p* = 0.15, HDI_Mean_  = 0.028, HDI_95%_  = [−0.00, 0.06]; κ_SELF_: M = 0.19; SD = 0.14; κ_OTHER_: M = 0.17; SD = 0.14) (Figure 4C−E). Bayesian analysis indicated anecdotal evidence for the null hypothesis (BF01 = 2.31). Similarly, for the model 2κ2β, the difference between κ self and κ other was not significant (*t*(46) = −0.78, *p* = 0.44, BF01 = 4.73; Figure S3C).

Participants’ effort discounting of the other's pain reduction showed a significant negative correlation with attitudes to helping (HAS score; *r* = 0.36, *t*(45) = 2.55, *p* = 0.014, BF01 = 0.297): individuals with greater positive attitudes toward helping discounted less the reduction of other's pain by effort. Discounting was not significantly correlated with trait cognitive (*r* = 0.01, *t*(45) = 0.10, *p* = 0.93, BF01 = 5.48) and affective (*r* = 0.04, *t*(48) = 0.24, *p* = 0.81, BF01 = 5.35) empathy.

### 3.4 Individuals Show Prosocial Motivation Despite Judging Others’ Pain as Less Intense and Unpleasant Than Their Own

**FIGURE 5:**
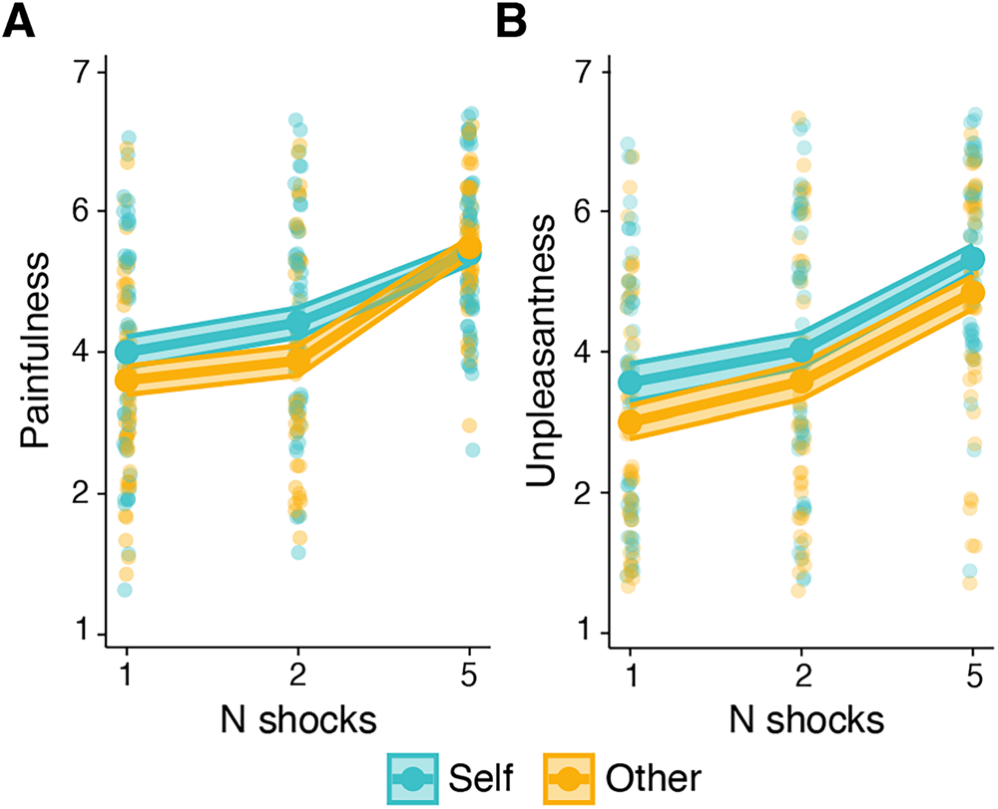
Ratings of shocks’ painfulness and unpleasantness. Ratings of shocks painfulness (A) and unpleasantness (B) as a function of recipient and number of shocks received. The bigger points represent the group means, and the smaller points represent individual ratings. The shaded ribbons represent the standard error of the mean.

Participants rated 1 and 2 (but not 5) shocks as more painful when delivered to themselves (1: M = 4.00, SD = 1.54; 2: M = 4.42, SD = 1.47; 5: M = 5.40, SD = 1.11) than when received by the other participant (1: M = 3.60, SD = 1.43; 2: M = 3.88, 1.48; 5: M = 5.49, SD = 0.92) (Recipient × N shocks: b = 0.32, CI [0.07, 0.57], *p* = 0.013; Figure 5A and Table S5). Further, they rated the shocks they received as more unpleasant (1: M = 3.56, SD = 1.89; 2: M = 4.02, SD = 1.74; 5: M = 5.32, SD = 1.38) than those received by the other participant (1: M = 3.00, SD = 1.65; 2: M = 3.58, SD = 1.81; 5: M = 4.84, SD = 1.64) (Recipient: b = ‐0.30, CI [‐0.53, ‐0.08], *p* = 0.008; Figure 5B and Table S6). Ratings of painfulness and unpleasantness of shocks received by the other person were not significantly correlated with the number of choices to exert effort (painfulness: *r* = 0.16, *t*(48) = 1.11, *p* = 0.27; unpleasantness: *r* = 0.12, *t*(48) = 0.80, *p* = 0.43) and force exerted (painfulness: *r* = 0.16, *t*(48) = 1.08, *p* = 0.28; unpleasantness: *r* = 0.13, *t*(48) = 0.88, *p* = 0.38) to help the other person, nor with the discount parameter for other (painfulness: *r* = 0.16, *t*(48) = 1.10, *p* = 0.28; unpleasantness: *r* = 0.19, *t*(45) = 1.35, *p* = 0.18).

To sum up, participants showed similar motivation to mobilize effort to reduce their and another person's pain, although they considered the other's pain as less intense and unpleasant than their own.

## 4 Discussion

Are humans willing to incur effort costs to alleviate others’ pain? And if they are, do they demonstrate equal motivation to protect others and themselves from harm, or does a self‐serving bias prevail? In contrast to the selfish tendencies observed by prior research using monetary rewards, our findings demonstrate that individuals are similarly motivated to incur effort costs to reduce their own pain and that of a stranger. Specifically, we found no credible evidence of a difference between self‐benefitting and prosocial behavior in terms of (1) probability of choosing to exert effort to reduce the number of delivered painful electric shocks, (2) response time to make such choices, (3) actual physical force exerted to achieve the shock reduction, and little credible evidence of a difference between devaluation of self‐ and other‐directed pain reduction with increasing effort (effort discounting).

These findings stand in stark contrast to previous studies, which indicated that individuals are prosocially apathetic and superficial when effort is required to help others [11−16]. Crucially, these studies examined effortful prosocial behavior in the context of financial outcomes, that is, gaining monetary rewards or avoiding monetary losses. We showed that, in the case of nonfinancial outcomes, that is, avoiding physical harm, individuals consider it equally worthwhile to incur effort costs to help a stranger as to help themselves. This raises the possibility that it may not be the type of cost—effort—that makes people less willing to help others, but rather the type of incentive at stake—increasing another's wealth—that does not sufficiently motivate effortful prosocial behavior. Given that prosocial apathy has been observed in monetary contexts involving both gaining money and avoiding financial loss [13], the asymmetry between egocentric and prosocial tendencies for these incentives cannot simply be explained by the appetitive (gain money) and aversive (avoid pain) nature of the paradigms employed. However, only future studies that directly compare the monetary and pain‐related incentives will be able to conclusively determine the validity of this interpretation.

Our findings are consistent with previous research showing that individuals learn more optimally [29] and are willing to incur greater monetary costs to avoid others’ pain than their own pain [23, 25, 26]. Additionally, they are more altruistic when allocating pain compared to money between themselves and another person in a dictator game [28]. Our study crucially extends these findings by demonstrating that this prosocial preference to protect others from harm not only occurs in the absence of costs (e.g., learning paradigms) or in the presence of moral and financial costs but also persists when effort expenditure is required to act prosocially. Compared to prior studies, which assessed only choice behavior, our findings also demonstrate that this prosocial preference encompasses action invigoration, as participants exerted a similar amount of effort in self and other trials.

As participants did not differ in their motivation to help themselves and a stranger, we did not observe “hyperaltruism” like in some of the previous studies focused on pain avoidance [23, 25, 29], but see Refs. [27, 28]. This is possibly due to methodological differences among the present and prior studies. For example, Lengersdorff et al. [29] examined implicit prosocial behavior (i.e., prosocial learning), which does not involve any explicit cost for the individual. Crockett et al. [23, 25] used a paradigm with a strong moral connotation, as individuals could choose whether to profit (gain more money) from harming others. In contrast to Crockett et al. [23] and Lockwood et al. [15], we also did not observe significant differences in response time between self‐ and other‐related decisions. This finding does not support the idea that prosocial choices are generated by faster, more intuitive processes, nor that they rely on slower, more deliberative ones compared to self‐benefitting choices [51, 52], but see Ref. [53].

Several explanations may account for the observed discrepancies in effortful prosocial motivation between the current study and those involving monetary outcomes. First, divergence in prosocial behavior in the monetary and pain contexts may arise from the contrasting valence of the outcomes involved (positive/appetitive for monetary gain and negative/aversive for pain avoidance). However, evidence from a previous study reporting selfish tendencies also in the context of avoiding monetary loss [13] makes this explanation unlikely. Second, greater prosociality in pain contexts may stem from individuals’ self‐centered view of being more resilient to pain than others [23]. Nevertheless, our participants rated the unpleasantness and intensity of the other's pain as lower and thus as less aversive than their own. Although these ratings reflected participants’ assumptions about the other person's pain, as no video of the other person receiving the shocks was shown, this suggests that participants’ prosocial tendencies are not merely driven by the belief that they can tolerate pain better than the other person. Third, empathy is a well‐known driver of prosocial behavior [54, 55]. Accordingly, we recently showed that the reduction in effortful behavior to reduce others’ pain following placebo analgesia is mediated by changes in empathy [30]. It is possible that individuals naturally empathize more with the physical suffering of others than with their financial gains or losses. However, in this study, we did not observe a significant association between prosocial behavior and empathic abilities (assessed by a trait questionnaire). This suggests that while empathy may play a role based on theoretical considerations, it might not fully explain the observed differences in prosocial behavior across different contexts. Fourth, previous research suggested that individuals might demonstrate greater prosociality due to uncertainty about the negative impact of the choice outcome on others [56]. It has been hypothesized that this would lead people to behave more cautiously to avoid unbearable outcomes for others and that this uncertainty effect is stronger in contexts of harm than in economic ones [23, 24, 28]. The results from the computational modeling analyses showed that participants’ choices were best described by a single noise parameter (β), suggesting a similar level of uncertainty during self‐benefitting and prosocial choices [57]. However, since our study was not designed to specifically assess impact uncertainty [58], future studies are needed to examine this hypothesis further. Fifth, interpersonal harm aversion, particularly in the case of physical pain, is a universal and deeply rooted social norm and is considered as one foundation of moral behavior [59, 60]. Thus, the moral weight associated with preventing harm to others may drive people to act more prosocially in harm‐related scenarios. The norm‐activation model [61, 62] posits that prosocial behavior follows from “personal norms” (i.e., feelings of moral obligation to help), which are influenced by the awareness of and the feelings of responsibility for the negative consequences of not helping. It is possible that avoiding physical harm to other, compared to increasing their wealth, is associated with stronger feelings of moral obligation. Last, although our sample is similar in age and gender distribution to those used in previous studies involving monetary incentives and prior research suggests people tend to be more altruistic in pain‐related contexts [23−29], we cannot entirely rule out the possibility that differences in prosocial motivation are due to unique characteristics of our sample.

Overall, further research that directly compares effortful prosocial motivation in pain and monetary contexts will be essential to provide conclusive evidence on the mechanisms underlying the observed discrepancies in prosocial motivation. Our findings offer preliminary insights into these mechanisms, providing direction for future research, and emphasize the need to move beyond an oversimplified dichotomy of humans being inherently selfish or altruistic. Instead, they highlight the importance of understanding the factors, particularly the type of benefit at stake, that drive individuals to prioritize their own or others’ interests in different situations.

### 4.1 Limitations

While this study provides valuable insights into the motivational processes underpinning effortful prosocial behavior, some limitations and open questions need to be noted. First, we did not directly compare effortful prosocial behavior for monetary and pain outcomes. Although the selfish tendencies when deciding whether to incur effort costs for monetary rewards have been replicated several times [11−17], future studies should compare effortful prosocial behavior in the harm and financial contexts within the same study sample. Second, we cannot exclude that the observed discrepancies between the pain and monetary contexts are driven by asymmetries in the subjective values attributed by the decider in relation to avoiding pain and gaining (or avoiding losing) money. Individuals may exhibit distinct prosocial tendencies in these two contexts simply because they are more motivated to increase their wealth than to avoid pain, in line with the *modality‐dependent egoistically biased altruism* described by Volz et al. [63]. This seems plausible also because most prior studies involved student samples, who are often primarily interested in the monetary compensation for their participation in an experiment. Studies using adequately matched monetary and pain outcomes will be crucial to exclude this possibility. We also acknowledge that in their original paradigms, Lockwood et al. [13] used abstract credits as outcomes, but we used the number of shocks instead, since treating physical pain as an abstract credit would be neither meaningful nor ethical. Nevertheless, in both paradigms, outcomes remain indirect, as shocks and money are physically delivered only after the task, not on a trial‐by‐trial basis. Third, while the best‐fitting model included separate discount parameters for self and other, the extracted subject‐level parameters were not statistically different in the two conditions. Although model comparison and parameter selection are different processes and can lead to different results, the findings from the computational modeling analyses should be interpreted cautiously. For enhanced transparency, we also reported model validation and parameter recovery for both the 2k2b and 2k1b models in the Supplementary Materials so that readers could comprehensively evaluate them. Fourth, power analysis was based on repeated‐measures *F*‐test, which approximates but does not fully match the assumptions of the gLMMs used in our main analyses. However, this pragmatic approach is widely accepted, and our main (null) findings were supported by Bayesian analyses, suggesting the study was adequately powered. Last, to minimize the effect of reciprocity and reputation, participants performed the task with a stranger and believed their choices would be private with respect to the receivers and experimenters (as confirmed by the post‐test control questions). As prosocial behavior is known to be affected by familiarity with the person in need [64, 65], future studies should examine whether individuals become more or less willing to exert effort to reduce the pain of more or less familiar others.

## 5 Conclusions

This study sheds light on the motivational processes underlying interpersonal harm avoidance and effortful prosocial behavior. We demonstrated that individuals are equally willing to exert physical effort to reduce pain to themselves and a stranger, thus challenging previous claims that individuals are (in general) prosocially apathetic and superficial when effort is required to help. Our findings offer two key insights for future research on prosocial behavior: first, they demonstrate that prosocial motivation to prevent harm is not limited to decision‐making, but also extends to the actual execution of helping behavior, as reflected in the exerted physical effort; second, they raise the possibility that the nature of the outcome may substantially impact individuals’ tendencies toward selfish and prosocial behavior.

## Author Contributions

CM: Conceptualization, methodology, software, formal analysis, visualization, investigation, data curation, project administration, funding, supervision, writing—original draft, writing—review and editing; LZ: Formal analysis, writing—review and editing; GS: Conceptualization, writing—review and editing; CL: Conceptualization, methodology, supervision, resources, funding, writing—review and editing.

## Conflicts of Interest

The authors declare no competing interests.

## Supporting information




**Supplementary Materials**: nyas70075‐sup‐0001‐SuppMatt.pdf
