# Supplementary material for: Avoiding Pain to Others Motivates Effortful Prosocial Behavior
Source: Ann N Y Acad Sci. 2025 Oct 1;1553(1):379–90. doi: 10.1111/nyas.70075 (PMC12645266; doi:10.1111/nyas.70075)
Supplement: Supplementary file 1 — Supplementary Materials: nyas70075‐sup‐0001‐SuppMatt.pdf [file NYAS-1553-379-s001.pdf]

Supplementary Information

Avoiding pain to others motivates effortful prosocial behavior reducing prosocial apathy

Claudia Massaccesi[1,2*], Lei Zhang[1,3,4,5], Giorgia Silani[6], Claus Lamm[1]

[1] *Department of Cognition, Emotion, and Methods in Psychology, Faculty of Psychology, University of Vienna, Vienna, Austria*
[2] *Department of Psychology, Section for Biological Psychology and Cognitive Neuroscience, University of Bielefeld, Bielefeld, Germany*
[3] *Centre for Human Brain Health, School of Psychology, University of Birmingham, Birmingham, UK*
[4] *Institute for Mental Health, School of Psychology, University of Birmingham, Birmingham, UK*
[5] *Centre for Developmental Science, School of Psychology, University of Birmingham, Birmingham, UK*
[6] *Department of Clinical and Health Psychology, Faculty of Psychology, University of Vienna, Vienna, Austria*

* Corresponding author:
Email: claudia.massaccesi@univie.ac.at
Address: Liebiggasse 5, 1010, Vienna, Austria

This document includes:

1. Results tables of main statistical analyses (choice, reaction times, force exerted, success rate)
   a. Table S1: gLMM of choice behavior
   b. Table S2: gLMM of reaction times (RTs)
   c. Table S3: gLMM of force exerted (area under the curve, AUC)
   d. Table S4: gLMM of success rate
2. Computational modeling with the Hierarchical Bayesian approach
   a. Results from model validation (posterior predictive check) and parameter recovery
   b. Figure S1: model validation
   c. Figure S2: parameter recovery (model 2κ1β)
   d. Figure S3: parameter recovery (model 2κ2β)
   e. Figure S4: Behavioral data of participants excluded from computational modeling analyses
3. Results tables of statistical analyses on the ratings of pain intensity and unpleasantness
   a. Table S5: gLMM shock painfulness ratings
   b. Table S6: gLMM shock unpleasantness ratings
4. Control analyses
   a. Table S7: Ratings of "physical demand", "effort" and "unpleasantness"
   b. Table S8: Effects of fatigue on choice behavior
   c. Table S9: Effects of fatigue on force exerted
   d. Table S10: Effects of fatigue on success rate
   e. Table S11: Analysis of force using max value
   f. Table S12: Effects of gender on ratings of shocks' painfulness
   g. Table S13: Effects of gender effects on ratings of shocks' unpleasantness
5. Information on shocks delivery at the end of the session

1. **Results tables of main statistical analyses (choice, reaction times, force exerted, success rate)**

Table S1. gLMM of choice behavior

**Choice**	glmmTMB(choice ~ recipient * effort * Shock reduction + (recipient + effort + Shock reduction + recipient:effort | ID), family = binomial)			
Predictors	*Odds Ratios*	*CI*	*Statistic*	*p*
(Intercept)	7.90	4.04 – 15.45	6.04	**<0.001**
Recipient	1.57	0.74 – 3.34	1.18	0.238
Effort	0.12	0.09 – 0.15	-15.69	**<0.001**
Shock reduction	4.07	3.10 – 5.36	10.05	**<0.001**
Recipient × Effort	1.09	0.80 – 1.48	0.54	0.586
Recipient × Shock reduction	0.86	0.71 – 1.05	-1.44	0.151
Effort × Shock reduction	0.67	0.58 – 0.78	-5.37	**<0.001**
Recipient × Effort × Shock reduction	0.91	0.75 – 1.11	-0.95	0.344
Marginal R^2 / Conditional R^2	0.384 / 0.806			

Table S2. gLMM of reaction times (RTs)

**RTs**	glmmTMB(RT ~ recipient * effort * Shock reduction + (recipient + Shock reduction + effort + recipient:effort + Shock reduction:effort | ID)					
Predictors	*Beta*	*std. Beta*	*CI*	*std. CI*	*z*	*p*
(Intercept)	1.23	0.01	1.17 – 1.30	-0.12 – 0.14	34.23	**<0.001**
Recipient	0.00	0.01	-0.04 – 0.03	-0.08 – 0.06	0.13	0.774
Effort	0.10	0.20	0.06 – 0.13	0.13 – 0.26	5.78	**<0.001**
Shock reduction	-0.03	-0.06	-0.05 – -0.01	-0.10 – -0.03	-3.08	**0.002**
Recipient × Effort	-0.01	-0.01	-0.02 – 0.04	-0.05 – 0.07	-0.51	0.741
Recipient × Shock reduction	-0.02	-0.04	-0.04 – 0.00	-0.07 – 0.00	-2.00	0.046
Effort × Shock reduction	0.05	0.09	0.03 – 0.06	0.06 – 0.13	4.85	**<0.001**
Recipient × Effort × Shock reduction	-0.01	-0.01	-0.02 – 0.01	-0.05 – 0.02	-0.80	0.512
Marginal R^2 / Conditional R^2	0.053 / 0.350					

Table S3. gLMM of force exerted (area under the curve, AUC)

**Force (AUC)**	glmmTMB(AUC force ~ recipient * effort* shock reduction + (recipient + effort | ID))					
Predictors	*Beta*	*std. Beta*	*CI*	*std. CI*	*z*	*p*
(Intercept)	0.52	-0.03	0.50 – 0.53	-0.13 – 0.06	77.94	**<0.001**
Recipient	0.00	0.01	-0.01 – 0.01	-0.04 – 0.06	0.24	0.811
Effort	0.09	0.74	0.09 – 0.10	0.68 – 0.81	23.41	**<0.001**
Shock reduction	0.00	0.03	0.00 – 0.01	0.01 – 0.05	2.44	**0.015**
Recipient × Effort	-0.00	-0.02	-0.01 – 0.00	-0.04 – 0.01	-1.10	0.273
Recipient × Shock reduction	-0.00	-0.01	-0.01 – 0.00	-0.04 – 0.01	-1.09	0.277
Effort × Shock reduction	-0.00	-0.01	-0.00 – 0.00	-0.03 – 0.01	-0.65	0.513
Recipient × Effort × Shock reduction	-0.00	-0.00	-0.00 – 0.00	-0.03 – 0.02	-0.18	0.857
Marginal R^2 / Conditional R^2	0.575 / 0.772					

Table S4. gLMM of success rate

**Success rate**	glmmTMB(success ~ recipient * effort* Shock reduction + (recipient + Shock reduction + effort + recipient:effort + recipient:Shock reduction + Shock reduction:effort | ID)			
Predictors	*Odds Ratios*	*CI*	*z*	*p*
(Intercept)	20.70	13.81 – 31.03	14.67	**<0.001**
Recipient	0.92	0.71 – 1.18	-0.66	0.506
Effort	0.31	0.24 – 0.41	-8.50	**<0.001**
Shock reduction	1.24	1.03 – 1.51	2.25	**0.024**
Recipient × Effort	1.08	0.82 – 1.42	0.55	0.580
Recipient × Shock reduction	0.88	0.68 – 1.14	-0.96	0.338
Effort × Shock reduction	1.02	0.83 – 1.26	0.18	0.856
Recipient × Effort × Shock reduction	0.95	0.72 – 1.25	-0.37	0.713
Marginal R^2 / Conditional R^2	0.193 / 0.445			

2. Hierarchical Bayesian Modeling

Results from model validation and parameter recovery

The simulated dataset generated from model 2κ1β closely resembles the original data (i.e., posterior predictive checks), demonstrating that the model effectively captures key behavioral patterns present in the actual data (Figure S1). In terms of parameter recovery, all group-level parameters were successfully retrieved, with the mean value of the simulated parameters falling within the 95% HDI of the true distribution (Figure S2A). At the individual subject-level, reliable parameter recovery was indicated by a strong correlation (all $r > 0.8$) between the simulated and actual parameters (Figure S2B). For completeness, we conducted model validation and parameter recovery also for the model 2κ2β. The model captured key behavioral patterns of the actual data (Figure S1), and both group-level and individual level parameters can be reliably recovered (Figure S3).

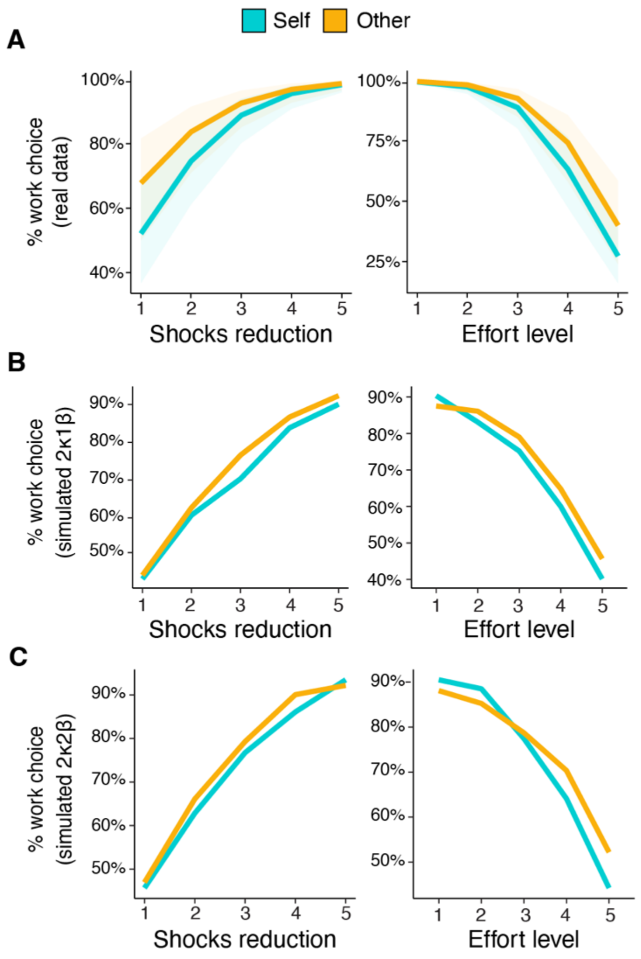

Figure S1. Model validation.
Average proportion of choosing the work option as a function of recipient (self, other), shocks reduction (left panels), and effort (right panels) for the actual data (A), the simulated data from the winning model 2κ1β (B), and the simulated data from model 2κ2β (C).

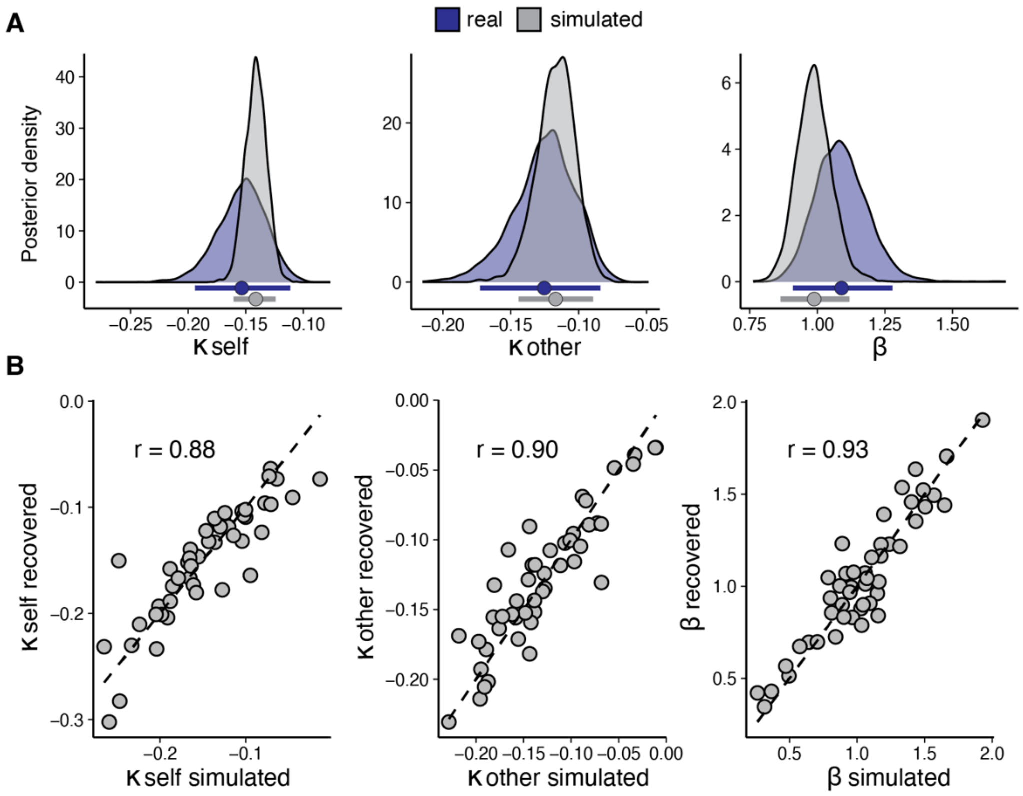

Figure S2. Parameter recovery (model 2κ1β).

(A) Parameter recovery of the group-level posterior distributions. Plots display the actual "ground truth" distribution (blue) and the distribution of the simulated dataset (grey) for each parameter. Horizontal bars represent the group-level 95% HDI of the parameter estimate, with dots indicating the mean of the distribution. (B) Parameter recovery of subject-level parameters. Plots depict Pearson correlations between actual recovered and simulated data for each parameter.

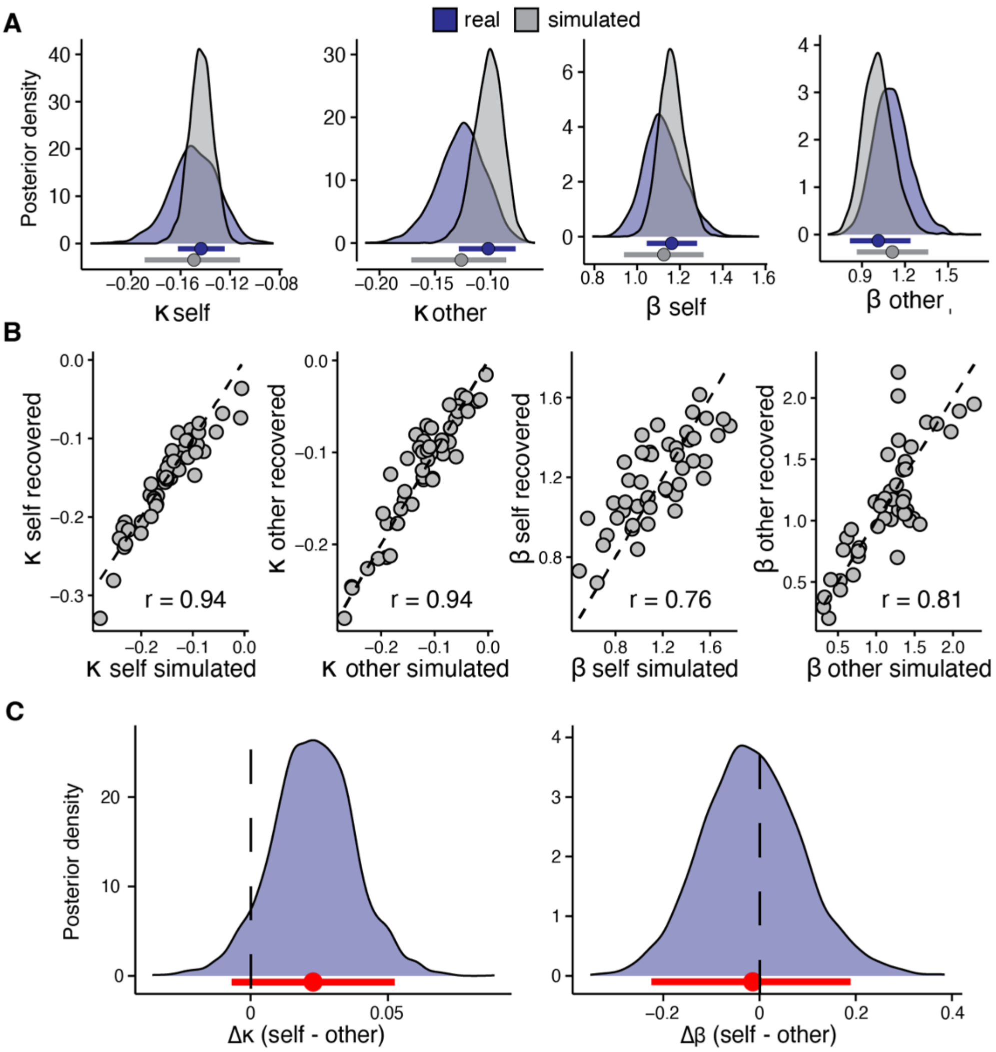

Figure S3. Parameter recovery (model 2κ2β) and self-other difference of group-level posterior distributions.

(A) Parameter recovery of the group-level posterior distributions. Plots display the actual "ground truth" distribution (blue) and the distribution of the simulated dataset (grey) for each parameter. Horizontal bars represent the group-level 95% HDI of the parameter estimate, with dots indicating the mean of the distribution. (B) Parameter recovery of subject-level parameters. Plots depict Pearson correlations between actual recovered and simulated data for each parameter. (C) Self-other difference of actual group-level posterior distributions of parameter κ (left panel) and β (right panel). Horizontal bars represent the group-level 95% HDI of the parameter estimate, with dots indicating the mean of the distribution.

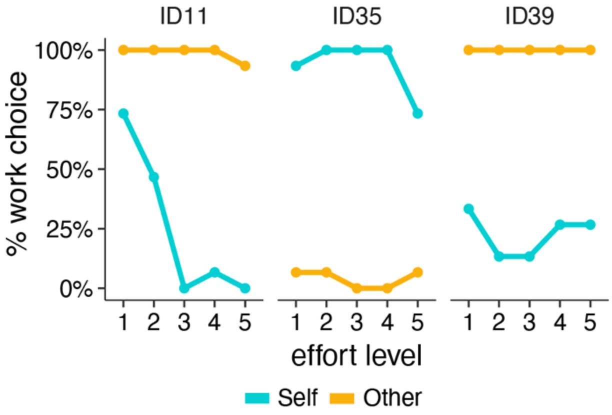

Figure S4. Behavioral data of participants excluded from computational modeling analyses. Average proportion of choosing the work option as a function of recipient (self, other) and effort of the three participants excluded from the computational modeling analyses (due to near-zero behavior variance in choice data).

3. Results tables of statistical analyses on the ratings of pain intensity and unpleasantness

Table S5. gLMM of shocks painfulness ratings

**Rating pain intensity**	glmmTMB(Rating painfulness ~ recipient * n_shocks + (recipient * n_shocks | ID))					
Predictors	*Beta*	*std. Beta*	*CI*	*std. CI*	*z*	*p*
(Intercept)	4.00	-0.31	3.58 – 4.42	-0.58 – -0.03	18.57	**<0.001**
Recipient	-0.40	-0.26	-0.80 – -0.00	-0.52 – -0.00	-1.98	**0.048**
N shocks [2]	0.42	0.28	0.06 – 0.78	0.04 – 0.51	2.32	**0.021**
N shocks [5]	1.40	0.92	1.09 – 1.71	0.72 – 1.12	8.89	**<0.001**
Recipient × N shocks [2]	-0.15	-0.10	-0.57 – 0.26	-0.37 – 0.17	-0.74	0.461
Recipient × N shocks [5]	0.49	0.32	0.10 – 0.87	0.07 – 0.57	2.48	**0.013**
Marginal R^2 / Conditional R^2	0.235 / 0.915					

Table S6. gLMM of shocks unpleasantness ratings

**Rating unpleasantness**	glmmTMB(Rating unpleasantness ~ recipient * n_shocks + (recipient * n_shocks | ID))					
Predictors	*Beta*	*std. Beta*	*CI*	*std. CI*	*z*	*p*
(Intercept)	3.56	-0.27	3.04 – 4.08	-0.54 – 0.01	13.48	**<0.001**
Recipient	-0.56	-0.30	-0.98 – -0.14	-0.53 – -0.08	-2.64	**0.008**
N shocks [2]	0.46	0.25	0.06 – 0.86	0.03 – 0.46	2.27	**0.023**
N shocks [5]	1.76	0.95	1.33 – 2.19	0.71 – 1.18	7.93	**<0.001**
Recipient × N shocks [2]	0.14	0.07	-0.30 – 0.58	-0.16 – 0.31	0.61	0.540
Recipient × N shocks [5]	0.10	0.05	-0.41 – 0.61	-0.22 – 0.33	0.38	0.702
Marginal R^2 / Conditional R^2	0.187	0.838				

4. Control analyses

Regarding the effort levels, participants' ratings of "physical demand", "effort" and "unpleasantness" roughly corresponded to what would be expected based on each level of effort (Table S7), indicating that the participants could distinguish between the different effort levels.

We also examined whether choices, force exerted, and success rate were affected by fatigue. To test for fatigue effects, we fitted all the mixed-effects models including the additional predictor "trial number". Despite the break every 50 trials, we found a significant effect of time (trial number) for all three variables, indicating that participants experienced a certain degree of fatigue during the task (Table S8, S9, S10). Nevertheless, no significant interactions between time and recipient (self, other) were found, indicating that the effects of fatigue on these variables were similar for self and other trials (Table S8, S9, S10). Therefore, participants did not change their prosocial behavior due to fatigue.

At the end of the session, we assessed whether participants believed their anonymity and their decisions were kept secret from the other participant, and whether they felt their decisions were monitored by the experimenters. Participants believed that their decisions and identity were kept secret from the other person (decisions: M = 4.6, SD = 0.78; identity: M = 4.8, SD = 0.45; rated from 1 "strongly disagree anonymity was kept" to 5 "strongly agree anonymity was kept") and felt minimally monitored during the study (M = 2.3, SD = 1.32; rated from 1 "I never felt monitored" to 5 "always felt monitored").

Table S7. Ratings of "physical demand", "effort" and "unpleasantness"

Effort required (% MVC)	Physical demand	Effort	Unpleasantness
**40**	20.54	24.48	40.48
**50**	40.38	35.68	48.80
**60**	51.60	44.90	60.76
**70**	64.16	56.80	72.44
**80**	77.68	70.22	84.26

Table S8. Effect of time on choice behavior

**Choice**	glmmTMB(choice ~ recipient * effort * shock reduction * trial + (recipient + effort + shock reduction + trial | ID), family = binomial)			
Predictors	*Odds Ratios*	*CI*	*Statistic*	*p*
(Intercept)	10.24	4.53 – 23.15	5.59	**<0.001**
Recipient	1.64	0.64 – 4.19	1.03	0.305
Effort	0.08	0.06 – 0.11	-15.28	**<0.001**
Shock reduction	5.26	3.83 – 7.22	10.28	**<0.001**
**Trial**	**0.58**	**0.42 – 0.79**	**-3.43**	**0.001**
Recipient × Effort	1.15	0.88 – 1.51	1.00	0.315
Recipient × Shock reduction	0.86	0.69 – 1.08	-1.29	0.197
Effort × Shock reduction	0.65	0.55 – 0.77	-5.01	**<0.001**
Recipient × Trial	0.97	0.78 – 1.21	-0.25	0.802
**Effort × Trial**	**0.52**	**0.43 – 0.62**	**-7.29**	**<0.001**
**Shock reduction × Trial**	**1.36**	**1.17 – 1.59**	**3.99**	**<0.001**
Recipient × Effort × Shock reduction	1.09	0.87 – 1.35	0.73	0.463
Recipient × Effort × Trial	0.90	0.72 – 1.14	-0.85	0.397
Recipient × Shock reduction × Trial	1.05	0.86 – 1.29	0.48	0.631
Effort × Shock reduction × Trial	0.91	0.78 – 1.06	-1.20	0.230
Recipient × Effort × Shock reduction × Trial	1.15	0.93 – 1.42	1.30	0.192
Marginal R^2 / Conditional R^2	0.394 / 0.866			

Table S9. Effects of time on force exerted

**Force (AUC)**	glmmTMB(AUC force ~ recipient * effort *shock reduction*trial + (recipient + effort + trial+ recipient:effort | ID))					
Predictors	*Beta*	*std. Beta*	*CI*	*std. CI*	*z*	*p*
(Intercept)	0.51	-0.03	0.50 – 0.53	-0.13 – 0.07	76.77	**<0.001**
Recipient	-0.00	-0.00	-0.01 – 0.01	-0.07 – 0.06	-0.24	0.812
Effort	0.09	0.74	0.09 – 0.10	0.68 – 0.80	24.72	**<0.001**
Shock reduction	0.00	0.03	0.00 – 0.01	0.02 – 0.05	3.67	**<0.001**
**Trial**	**-0.01**	**-0.05**	**-0.01 – -0.00**	**-0.10 – -0.01**	**-2.24**	**0.025**
Recipient × Effort	-0.00	-0.03	-0.01 – 0.00	-0.08 – 0.02	-1.18	0.238
Recipient × Shock reduction	-0.00	-0.03	-0.01 – 0.00	-0.05 – -0.00	-1.80	0.071
Effort × Shock reduction	-0.00	-0.00	-0.00 – 0.00	-0.02 – 0.02	-0.12	0.905
Recipient × Trial	0.00	0.01	-0.00 – 0.00	-0.01 – 0.04	0.41	0.685
Effort × Trial	0.00	0.01	-0.00 – 0.00	-0.01 – 0.03	0.73	0.466
Shock reduction × Trial	0.00	0.01	-0.00 – 0.00	-0.00 – 0.03	1.75	0.081
Recipient × Effort × Shock reduction	0.00	0.00	-0.00 – 0.00	-0.02 – 0.03	0.28	0.778
Recipient × Effort × Trial	-0.00	-0.02	-0.01 – 0.00	-0.05 – 0.00	-1.87	0.061
Recipient × Shock reduction × Trial	-0.00	-0.00	-0.00 – 0.00	-0.03 – 0.02	-0.05	0.957
Effort × Shock reduction × Trial	0.00	0.01	-0.00 – 0.00	-0.01 – 0.03	0.92	0.357
Recipient × Effort × Shock reduction × Trial	0.00	0.01	-0.00 – 0.00	-0.01 – 0.03	0.77	0.442
Marginal R^2 / Conditional R^2	0.570 / 0.803					

Table S10. Effect of time on success rate

**Success rate**	glmmTMB(success rate ~ recipient * effort *shock reduction*trial + (recipient + effort + trial| ID))			
Predictors	*Odds Ratios*	*CI*	*z*	*p*
(Intercept)	20.28	13.41 – 30.67	14.26	**<0.001**
Recipient	0.98	0.69 – 1.38	-0.11	0.911
Effort	0.30	0.23 – 0.40	-8.38	**<0.001**
Shock reduction	1.28	1.06 – 1.56	2.51	**0.012**
**Trial**	**0.78**	**0.62 – 0.98**	**-2.11**	**0.035**
Recipient × Effort	1.00	0.73 – 1.39	0.03	0.977
Recipient × Shock reduction	0.88	0.67 – 1.16	-0.92	0.357
Effort × Shock reduction	1.04	0.84 – 1.28	0.35	0.726
Recipient × Trial	1.01	0.78 – 1.32	0.10	0.923
Effort × Trial	1.02	0.82 – 1.26	0.18	0.859
Shock reduction × Trial	1.04	0.86 – 1.25	0.37	0.710
Recipient × Effort × Shock reduction	0.99	0.74 – 1.33	-0.04	0.968
Recipient × Effort × Trial	0.90	0.68 – 1.18	-0.78	0.438
Recipient × Shock reduction × Trial	0.92	0.71 – 1.20	-0.60	0.550
Effort × Shock reduction × Trial	0.96	0.77 – 1.19	-0.40	0.693
Recipient × Effort × Shock reduction × Trial	1.12	0.84 – 1.48	0.77	0.442
Marginal R^2 / Conditional R^2	0.204 / 0.478			

Table S11. Analysis of force using max value. For completeness, we also conducted the analysis on force exerted using the max force value in the 3s window instead of the area under the curve. The two analyses resulted in comparable findings.

**Force (Max)**	glmmTMB(Max force ~ recipient * effort* shock reduction + (recipient + effort | ID))					
Predictors	*Beta*	*std. Beta*	*CI*	*std. CI*	*z*	*p*
(Intercept)	176.01	0.05	159.06 – 192.96	-0.21 – 0.30	0.35	**<0.001**
Recipient	0.33	0.01	-1.15 – 1.82	-0.02 – 0.03	0.56	0.660
Effort	31.14	0.47	27.58 – 34.70	0.41 – 0.52	17.13	**<0.001**
Shock reduction	0.10	0.00	-0.50 – 0.70	-0.00 – 0.01	0.93	0.747
Recipient × Effort	-0.47	-0.01	-1.30 – 0.37	-0.02 – 0.01	-1.08	0.275
Recipient × Shock reduction	-0.30	-0.00	-1.11 – 0.51	-0.02 – 0.01	-0.81	0.472
Effort × Shock reduction	-0.60	-0.01	-1.21 – -0.00	-0.02 – -0.00	-1.96	**0.050**
Recipient × Effort × Shock reduction	0.07	0.00	-0.75 – 0.89	-0.01 – 0.01	0.17	0.865
Marginal R^2 / Conditional R^2	0.185 / 0.960					

Table S12. Effects of gender on ratings of shocks' painfulness

**Rating pain intensity**	glmmTMB(Rating painfulness ~ recipient * n_shocks * gender + (recipient * n_shocks | ID))					
Predictors	*Estimates*	*std. Beta*	*CI*	*std. CI*	*z*	*p*
(Intercept)	4.18	-0.30	3.55 – 4.81	-0.58 – -0.03	12.95	**<0.001**
Recipient	-0.55	-0.26	-1.14 – 0.05	-0.52 – -0.00	-1.80	0.072
N shocks [2]	0.45	0.28	-0.08 – 0.99	0.04 – 0.51	1.66	0.096
N shocks [5]	1.27	0.92	0.81 – 1.74	0.72 – 1.12	5.39	**<0.001**
gender	-0.32	-0.11	-1.17 – 0.52	-0.38 – 0.17	-0.75	0.452
Recipient × N shocks [2]	-0.14	-0.11	-0.74 – 0.47	-0.37 – 0.16	-0.44	0.658
Recipient × N shocks [5]	0.56	0.32	-0.02 – 1.15	0.07 – 0.57	1.90	0.058
Recipient × gender	0.26	0.08	-0.53 – 1.05	-0.17 – 0.34	0.64	0.522
N shocks [2] × gender	-0.06	-0.02	-0.78 – 0.65	-0.25 – 0.21	-0.17	0.866
N shocks [5] × gender	0.23	0.07	-0.39 – 0.85	-0.13 – 0.28	0.72	0.472
Recipient × N shocks [2] × gender	-0.04	-0.01	-0.85 – 0.77	-0.28 – 0.25	-0.10	0.918
Recipient × N shocks [5] × gender	-0.14	-0.04	-0.91 – 0.64	-0.30 – 0.21	-0.34	0.733
Marginal R^2 / Conditional R^2	0.241 / 0.915					

Table S13. Effects of gender on ratings of shocks' unpleasantness

**Rating unpleasantness**	glmmTMB(Rating unpleasantness ~ recipient * n_shocks * gender + (recipient | ID))					
Predictors	*Estimates*	*std. Beta*	*CI*	*std. CI*	*z*	*p*
(Intercept)	3.77	-0.26	3.08 – 4.47	-0.51 – -0.01	10.61	**<0.001**
Recipient	-0.77	-0.30	-1.49 – -0.05	-0.56 – -0.04	-2.11	**0.035**
N shocks [2]	0.23	0.25	-0.39 – 0.85	0.03 – 0.47	0.72	0.472
N shocks [5]	1.55	0.95	0.93 – 2.16	0.73 – 1.17	4.89	**<0.001**
gender	-0.38	-0.10	-1.31 – 0.55	-0.35 – 0.15	-0.80	0.424
Recipient × N shocks [2]	0.32	0.05	-0.56 – 1.19	-0.26 – 0.37	0.71	0.476
Recipient × N shocks [5]	-0.13	0.06	-1.01 – 0.75	-0.26 – 0.37	-0.29	0.775
Recipient × gender	0.38	0.10	-0.58 – 1.34	-0.16 – 0.36	0.78	0.438
N shocks [2] × gender	0.42	0.11	-0.41 – 1.24	-0.11 – 0.33	0.99	0.325
N shocks [5] × gender	0.38	0.10	-0.44 – 1.21	-0.12 – 0.32	0.91	0.364
Recipient × N shocks [2] × gender	-0.39	-0.10	-1.56 – 0.78	-0.42 – 0.21	-0.65	0.514
Recipient × N shocks [5] × gender	0.41	0.11	-0.76 – 1.59	-0.20 – 0.43	0.69	0.489
Marginal R^2 / Conditional R^2	0.198 / 0.681					

5. Information on shocks delivery at the end of the session

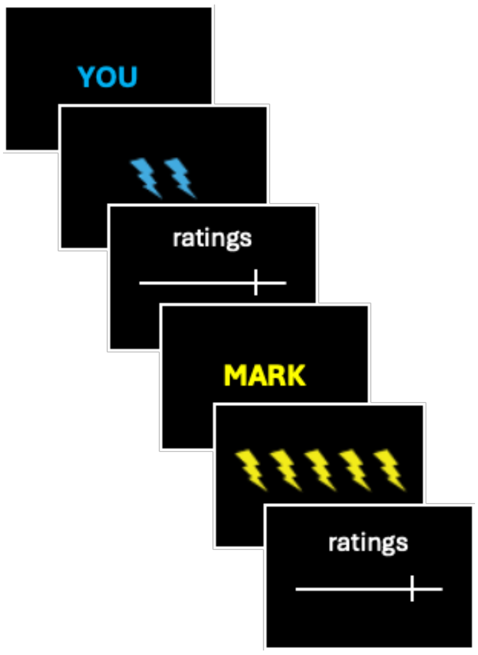

Figure S5. Shocks delivery at the end of the session. After the task, three trials were (allegedly) randomly selected and paid out (i.e., the shocks were delivered) to each recipient. In reality, trials with the same three outputs were always selected for both recipients (1, 2, and 5 shocks, presented in a randomized fashion) and the confederate never received any shock. After each outcome was paid out, the participant was asked to rate the painfulness and unpleasantness of the shocks received by themselves ("How painful were these shocks for you?"; "How unpleasant was it for you to receive these shocks?") and by the confederate ("How painful were these shocks for the other participant?"; "How unpleasant was it for you when the other participant received these shocks?"), on a 7-point Likert scale (1 = not at all, 7 = extremely). Participants were instructed to guess the other's pain, no video of the confederate receiving the shocks was shown.
